# Characterization and Preparation of Nanostructured Al_2_Fe_3_Si_3_ Thermoelectric Materials

**DOI:** 10.3390/ma18225193

**Published:** 2025-11-15

**Authors:** Zhiyang Zhao, Teruyuki Ikeda

**Affiliations:** Graduate School of Science and Engineering, Ibaraki University, 4-12-1 Nakanarusawa, Hitachi 316-8511, Ibaraki, Japan; 23nd153s@vc.ibaraki.ac.jp

**Keywords:** nanostructure, oxide particles, composite, thermoelectric properties

## Abstract

A nanostructured Al_2_Fe_3_Si_3_ composite has been synthesized by high-energy ball milling followed by spark plasma sintering. Formations of oxides and FeSi or Al_2_Fe_3_Si_4_ phases in nanometer-size scales were observed in addition to the matrix Al_2_Fe_3_Si_3_ phase. Because of enhanced phonon scattering, the materials show low lattice thermal conductivities such as ~5 W/mK at the minimum.

## 1. Introduction

Thermoelectric (TE) materials can be used for heat recovery because they can realize the conversion from heat energy to electrical energy. Recently, in accordance with the development of the internet of things (IoT), expectations have been heightened for TE devices to be used as autonomous power supplies for IoT devices [1] due to the silent operative and maintenance-free characteristics of thermoelectric power generators. Fe-Al-Si thermoelectric (FAST) materials are promising materials for autonomous power supplies for IoT devices because of their nontoxic, inexpensive, and abundant constituent elements. As previously reported [2], FAST materials were compared to the Bi-Te-based TE materials, which are commonly used at room temperature. Although the power density of FAST materials was approximately one-fifth that of Bi-Te-based materials, the cost payback time and robustness of FAST materials is superior to that of Bi-Te-based materials. While FAST material is being studied for practical use [3], improving the thermoelectric properties of the material is still important because the higher performance of the material will lead to a widened range of the sensors which can be driven by the power supplies or to the making of more compact modules.

Thermoelectric conversion efficiency depends on the dimensionless figure of merit *zT* = *S*^2^σ*T*/(κ_ele_ + κ_lat_), where *S*, σ, κ_ele_, and κ_lat_ are the Seebeck coefficient, electrical conductivity, electronic thermal conductivity, and lattice thermal conductivity, respectively [4,5,6]. Numerous studies have been carried out for the purpose of reducing lattice thermal conductivity via nanostructuring and alloying [7,8,9], etc. Nanostructuring, in general, enhances phonon scattering by increasing the frequency for phonons to encounter boundaries [10,11,12]. Alloying enhances phonon scattering by introducing mass contrast between neighboring atoms or strains in atomistic scales. We consider here what strategy should be taken to reduce the thermal conductivity of the FAST materials. FAST materials are composed only of abundant elements in the Earth’s crust and hence alloying with impure elements, especially heavy elements which could be most effective in light of phonon scattering, could spoil the materials’ advantage. While alloying effects with phonon scattering could be introduced in controlling the off-stoichiometry, that is, the Al/Si ratio, one cannot control the Al/Si ratio only for achieving the lowest thermal conductivity, since carrier concentration also depends on the Al/Si ratio. Furthermore, phonons scattered by the alloying effect are limited to the phonons with relatively short mean free paths, and hence there should be a possibility of reducing thermal conductivity by enhancing scattering phonons with longer mean free paths by nanostructuring [13,14].

The nanostructuring of FAST materials have not yet been studied. In this study, we synthesized nanostructured τ_1_-Al_2_Fe_3_Si_3_ to reduce κ_lat_. For the Al-Fe system, a ball-milling method has been employed to prepare nanostructured samples with improved mechanical properties [15]. In this system, it has been reported that the average particle size can be reduced to ~10 nm or even smaller. Such fine microstructures could effectively enhance phonon scattering, resulting in reduced thermal conductivity.

In this paper, we milled powders in various conditions to control the volume fraction of oxides, to eventually achieve nanometer-size scales. τ_1_-Al_2_Fe_3_Si_3_ compounds were prepared via spark plasma sintering after ball milling. Lowered thermal conductivities were observed in all samples, and minimum thermal conductivity, ~5 W/mK, at room temperature was achieved.

## 2. Materials and Methods

Powders of the raw materials, comprising Fe (<150 μm, 99%, Kojundo Chemical Lab. Co., Ltd., Saitama, Japan), Al (<150 μm, 99.99%, Kojundo Chemical Lab. Co., Ltd., Saitama, Japan), and grains of Si (2~5 mm, 99.999%, Kojundo Chemical Lab. Co., Ltd., Saitama, Japan) were weighed for prepared compositions of Al_25.7_Fe_37.1_Si_37.1_ (4%Al), Al_26.8_Fe_36.6_Si_36.6_ (10%Al), and Al_27.5_Fe_36.2_Si_36.2_ (14%Al), where 4, 10, and 14 at. % extra quantities of Al were added to the nominal composition of Al_25_Fe_37.5_Si_37.5_ to compensate for Al loss due to evaporation and oxidization during sintering at a later stage.

Pellet samples were prepared by two different routes; while samples with larger grains were prepared by a reactive sintering method [16], those with smaller grains with nanometer-size scales were prepared by the consolidation of powder mechanically alloyed by high-energy ball milling.

For reactive sintering, first, Si was milled with ZrO_2_ balls in a ZrO_2_ vial at 700 rpm for 2 h using a planetary ball mill (Planet-min, Nagao System Inc., Kanagawa, Japan) to obtain a fine powder. The Si powder was mixed with Fe powder at 550 rpm for 1.75 h using the same machine, then Al powder was added and mixed at 500 rpm for 1.25 h. In these steps, the lower rates of rotation were employed to mix the powders of different elements well but not to alloy them [16]. All operations were performed in an Ar atmosphere in a glove box equipped with a gas circulation purification system (UL-1000A, UNICO Ltd., Ibaraki, Japan). The mixed powder was loaded into a graphite die with a 12.4 mm inner diameter and sintered by hot pressing (HP) (SPD4000-I, Dai-ichi Kiden Co., Ltd., Tokyo, Japan) at 1223 K under a pressure of 84 MPa for 2 h under an argon atmosphere.

For preparing samples via high-energy ball milling, the powders of Al, Fe, and Si were milled with WC balls in a WC vial filled with argon gas in the glove box, where a viton O-ring was used to achieve better sealing for 70 h using a high-energy ball mill (Spex Mill 8000M, SPEX SamplePrep Corp., Metuchen, NJ, USA). For the No. 2 sample, while these operations were performed in air, the samples were repacked in the vial under Ar gas every 12 h in the glove box and the O-ring was replaced with a brand-new one every 30 h. For the No. 3 sample, the milling was performed in a WC vial sealed with Ar gas in a nylon bag for lower oxygen partial pressure than the No. 2 sample. Samples No. 4, 5, and 6 were milled in an Ar atmosphere in a glove box, where the oxygen level of the atmosphere was less than the detection limit of the zirconia-type oxygen analyzer of 0.001 ppm. The sample preparation conditions and bulk densities are summarized in Table 1.

The milled powder was loaded into a graphite die with a 12.4 mm inner cylindrical diameter and sintered by spark plasma sintering (SPS) (SPD4000-I, Dai-ichi Kiden Co., Ltd., Tokyo, Japan) at 1223 K under a pressure of 84 MPa for 10 min in an argon atmosphere. After sintering, the thickness and weight of the sintered body were measured to calculate the bulk density.

The crystal structures were characterized via X-ray diffraction analysis (XRD) using Cu Kα radiation (UtimaIV, Rigaku Corp., Tokyo, Japan). The microstructure was observed using a scanning electron microscope combined with a focused ion beam (FIB-SEM) (AurigaLaser, Carl Zeiss Co., Ltd., Oberkochen, Germany), and the composition of samples was measured by energy-dispersive spectroscopy (EDS) (EMAX7693-H, HORIBA Corp., Kyoto, Japan). Oxygen concentrations were measured with an inert gas-fusion elemental analyzer (ONH836, LECO Corp., St. Joseph, MI, USA). The Seebeck coefficient and electrical conductivity were measured using a Seebeck coefficient/electric resistance measurement system (ZEM-3, Advance Riko, Inc., Kanagawa, Japan) simultaneously. The thermal diffusivity, α, and heat capacity, *C_p_*, were measured by the laser flash method (LFA 447 Nanoflash, NETZSCH Japan K.K., Kanagawa, Japan). The thermal conductivity, κ_tot_, was calculated by κ_tot_ = αρ*C_p_*, where ρ is the bulk density of the sample and *C_p_* is the heat capacity.

## 3. Results and Discussion

**Figure 1:**
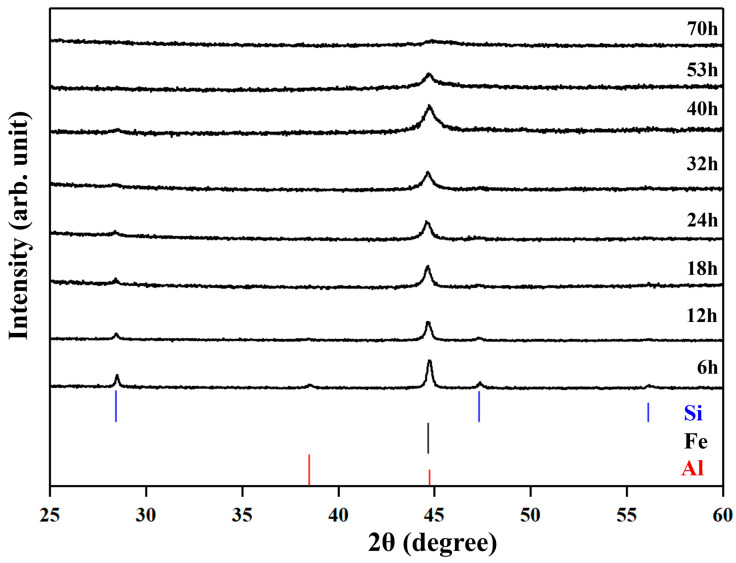
The XRD patterns of the No.3 sample during high-energy ball milling.

The XRD patterns of the No. 3 sample during the milling are shown in Figure 1. The peaks are blunter for longer milling times. At 70 h, almost no peaks are recognized, suggesting that the crystallite diameters are less than 10 nm or samples are in an amorphous state.

**Figure 2:**
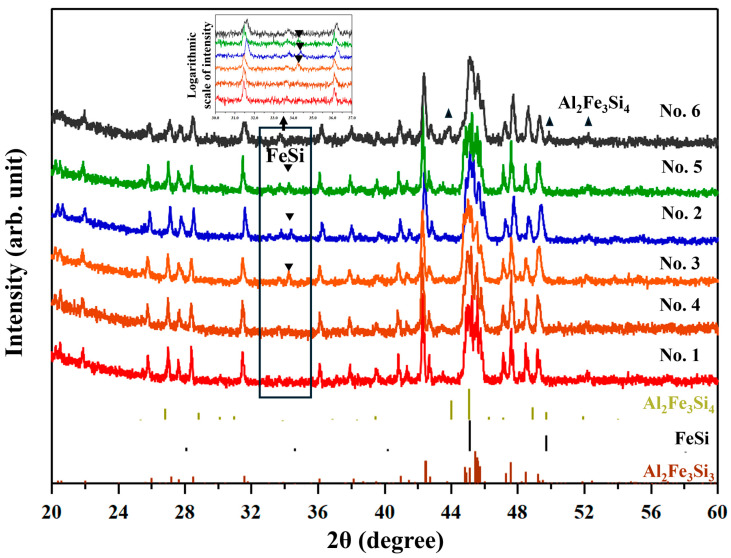
The XRD patterns of all samples after consolidation. Reference peaks from Al_2_Fe_3_Si_3_, ε-FeSi, and Al_2_Fe_3_Si_4_ are shown at the bottom. The inverted triangle symbol is the peak of ε-FeSi. The equilateral triangle symbol is the peak of Al_2_Fe_3_Si_4_. The profiles are enlarged in the small window shown at the top for 30–37° of 2q.

Figure 2 shows the XRD patterns of all samples after consolidation (SPS/HP). All samples have diffraction peaks which are identified as ones from the τ_1_-Al_2_Fe_3_Si_3_ phase. The No. 1–5 samples have an additional peak at around 35°, which is indexed as the ε-FeSi phase, similarly to previously reported samples [17,18,19]. The No. 6 sample has a peak near 43°, which is identified as that from the τ_8_-Al_2_Fe_3_Si_4_ phase [20]. According to the reference pattern of ε-FeSi of the ICDD database, there should be a second-strongest peak at around 49°. However, this peak is not clearly observed in the profiles of the current samples. The reason we deduced is that since the compositions of the ε-FeSi phase (containing Al in this study) and the t_1_-Al_2_Fe_3_Si_3_ phase (composition shifted from the stoichiometry) are different from the materials for the reference XRD patterns, the peak angles in the measured XRD profiles shift from the reference data. Actually, according to a previous work [21], doping Al to ε-FeSi shifts the peak to a lower angle by 0.2–0.4°. This causes the overlapping with the peak from t_1_-Al_2_Fe_3_Si_3_. The presumption that the secondary phase is ε-FeSi will be confirmed later in this paper with the aid of SEM observations and EBSD analysis. Other peaks from the ε-FeSi phase or τ_8_-Al_2_Fe_3_Si_4_ phase overlap with peaks of the τ_1_-Al_2_Fe_3_Si_3_ phase (see Figure S3 in Supplementary Materials for details). The diffraction peaks of oxides are not observed.

**Figure 3:**
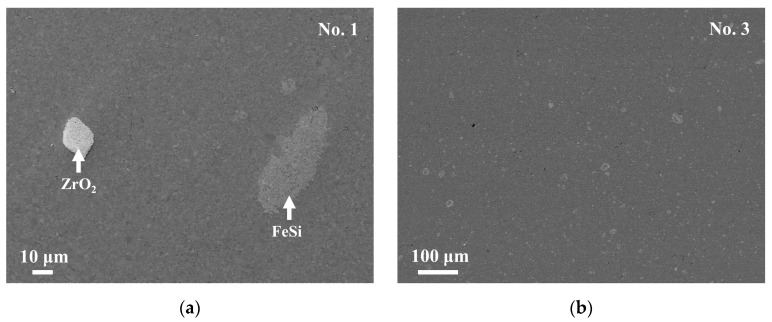
Backscattered electron (BSE) images of samples No. 1 (**a**) and No. 3 (**b**) after sintering. In No. 3 (**b**), several “bright phase” particles which seem to have diameters larger than 10 µm are actually hollow circles, where the insides are of the Al_2_Fe_3_Si_3_ phase and the edges are of the FeSi phase. Thus, the FeSi phase has a width of only less than 1 µm. The bright regions are of the ε–FeSi or the ZrO_2_ phase.

Figure 3a,b show the SEM images of the No. 1 and No. 3 samples. The chemical compositions of the respective phases obtained by EDS are shown in Table 2. The results of XRD, SEM, and EDS suggest that the matrix phase of both the samples is the τ_1_-Al_2_Fe_3_Si_3_ phase. The bright phase is recognized in the No. 3 sample, as shown in Figure 3b, but is too fine to be clearly analyzed for its chemical composition. The chemical composition will be discussed in the following section. In addition, the ZrO_2_ phase, which is from the vial and balls during the milling process, is also observed, as shown in Figure 3a.

**Figure 4:**
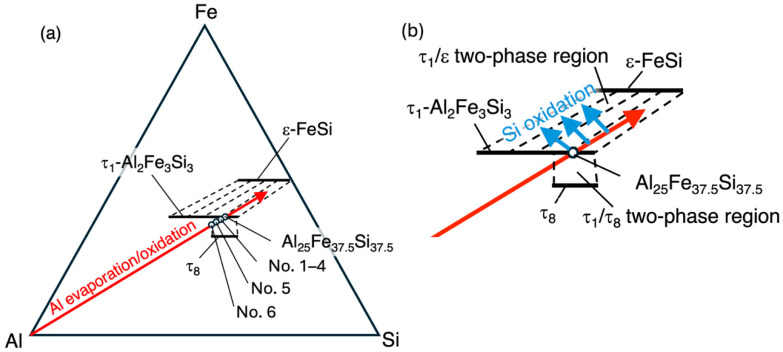
The charging compositions and their variation during synthesis. The charging compositions are schematically shown as solid circles aligned on the arrow connecting Al and Al_25_Fe_37.5_Si_37.5_ in the AlFeSi phase diagram [20] in (**a**). See the main text for the meaning of the arrow “Al evaporation/oxidation.” The region around the t_1_ phase is enlarged in (**b**). If silicon atoms in the samples are consumed by oxidation, the average composition of the unoxidized regions will shift in the directions shown as “Si oxidation”.

The charging compositions of all the samples No. 1–6, which are schematically shown in Figure 4a, were chosen so that the resulting compositions of the τ_1_ phase would become Al_25_Fe_37.5_Si_37.5_, supposing an Al loss of 4% for No. 1–4, 10% for No. 5, and 14% for No. 6, respectively, due to the evaporation or oxidation of Al during synthesis. These compositions are aligned on the line connecting Al and Al_25_Fe_37.5_Si_37.5_ (hereafter called the “Al loss line”) in the composition triangle of the Al-Fe-Si system.

Recently, the tie lines of the Al-Fe-Si system have been experimentally studied by the sintered diffusion multiple method [22]. According to that work, the tie lines between the τ_1_ phase and the ε phase (shown as broken lines in Figure 4b) are nearly parallel to the “Al loss line,” which is shown as the arrow connecting Al and Al_25_Fe_37.5_Si_37.5_. Eventually, as long as the average compositions of the unoxidized regions go in the direction of the two-phase region between the τ_1_ phase and the ε phase due to the loss of Al during synthesis, the samples will be composed of the τ_1_ phase and the ε phase, and the resulting Al/Si ratios of the τ_1_ phase in the samples will not vary much. The loss of Al can occur by the evaporation of Al, which causes the shift in the gross composition, or by the oxidization of Al, which causes the shift in the average composition of the unoxidized regions. Actually, samples No. 1–5 are composed of the τ_1_ and ε phases, and their Al/Si ratios do not differ very much.

In addition to Al loss, oxidation of Si can occur, resulting in the average composition of unoxidized regions being shifted in the directions shown by the arrows going away from Si in the composition triangle, with the Al-richer compositions of the τ_1_ phase in samples of Al_25_Fe_37.5_Si_37.5_ being to some extent dependent on conditions.

The No. 6 sample, which has the highest Al content, is composed of the τ_1_ and τ_8_ phases of the Al-Fe-Si system. This suggests that the “Al loss line” did not reach the two-phase region between the τ_1_ phase and the ε phase because of the high Al content but did go in the direction of the two-phase region between the τ_1_ and τ_8_ phases.

**Figure 5:**
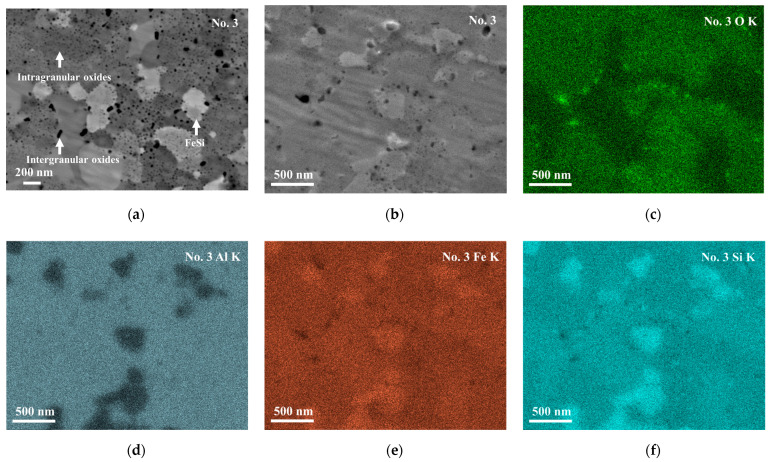
Microstructure of the No. 3 sample; BSE image (**a**), secondary electron (SE) image (**b**), and EDS mapping for oxygen K_a_ (**c**), aluminum K_a_ (**d**), iron K_a_ (**e**), and silicon K_a_ (**f**), respectively. In (**a**), relatively light regions and blank spots are of the FeSi phase and of oxides, respectively. The blank spots in (**b**) are rich in oxygen, as shown in (**c**).

Figure 5a,b show a backscattered electron (BSE) image and secondary electron (SE) image of the No. 3 sample with a high magnification. For the bright phase in Figure 5b, which corresponds to the bright phase in Figure 3b, it is difficult to determine its exact chemical composition by EDS because its size-scale is less than 1 µm. Looking into the elemental mapping data, as shown Figure 5c–f, it is found that the bright phase is rich in iron and silicon. From these data, together with the XRD profile (Figure 2) and electron backscatter diffraction pattern (EBSD) (Figure S4) image which show the existence of the ε-FeSi phase, and the reported phase diagram of the Al-Fe-Si phase [20], the bright phase can be concluded to be ε-FeSi.

The sizes of ε-FeSi particles have been determined by analyzing SEM images using the ImageJ (version 1.54g) software for the No. 3 sample. The particle diameter distribution is shown in Figure S5. The mean diameter is $\bar{d}$ = 0.23 μm, which is one order of magnitude smaller than those prepared by gas-atomization and SPS ($\bar{d}$ = 1.7 μm) [23]. Thus, as suggested previously [24], MA is more advantageous for realizing fine microstructures with nanometer-size scales.

The intragranular and intergranular black spots are observed in the No. 2–6 samples, similarly to those shown for No. 3 in Figure 5a,b. They are found to have nanometer scales, while there are not so many similar black spots found in the No. 1 sample, as shown in Figure S1. Figure 5c–f show the oxygen, aluminum, iron, and silicon distributions, respectively, in the No. 3 sample of the same region as in Figure 5b. The black spots in the SE image (Figure 5b) show high concentrations of oxygen (Figure 5c) and low concentrations of iron (Figure 5e) and silicon (Figure 5f). On the other hand, the aluminum (Figure 5d) mapping does not show contrast at the same positions as the SE image. This suggests that the black spots are of aluminum oxide, while the oxides are too small to be analyzed for their exact chemical compositions. The aluminum concentration in the τ_1_-Al_2_Fe_3_Si_3_ phase is ~25 at. % while that in aluminum oxide is ~40 at. %, assuming that the oxides are of Al_2_O_3_. Since the difference in aluminum concentration is not very large and the size of the particles is smaller than the spatial resolution of EDS analysis, no clear contrast can be observed in the aluminum mapping (Figure 5d).

The results of EDS in Table 2 show that the oxygen concentrations of the No. 1–3 samples depend on the atmosphere in milling; they increase with the oxygen content of the atmosphere. In other words, the oxygen content, i.e., the volume fraction of the small dots of oxide, is controlled by the oxygen content of the atmosphere in milling. Since it is known that the concentrations of light elements determined by EDS are, in general, not necessarily accurate [25], we double-checked the oxygen content of the No. 2 sample with inert gas-fusion elemental analyzers, and it is found to be 1.5 wt.% instead of the 5.3 wt.% found by EDS.

**Figure 6:**
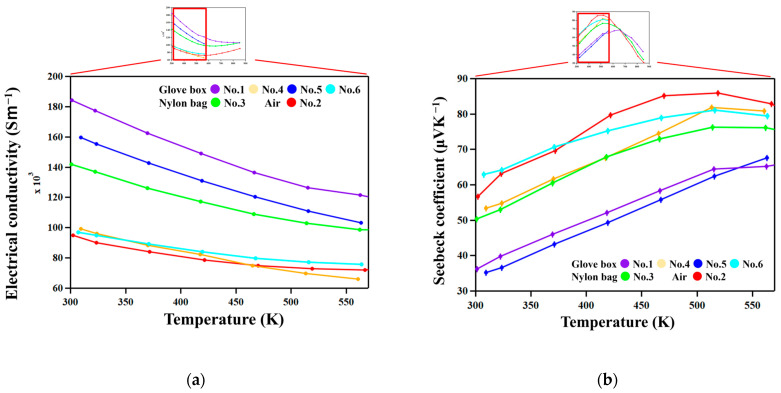
(**a**) The electrical conductivities as functions of temperature. (**b**) The Seebeck coefficients as functions of temperature.

Figure 6 shows the temperature dependence of the electrical conductivity σ and Seebeck coefficient *S*. All samples have positive Seebeck coefficients throughout the entire temperature range. σ decreased and *S* increased with increasing temperature in all the samples, exhibiting degenerate semiconductor behavior.

**Figure 7:**
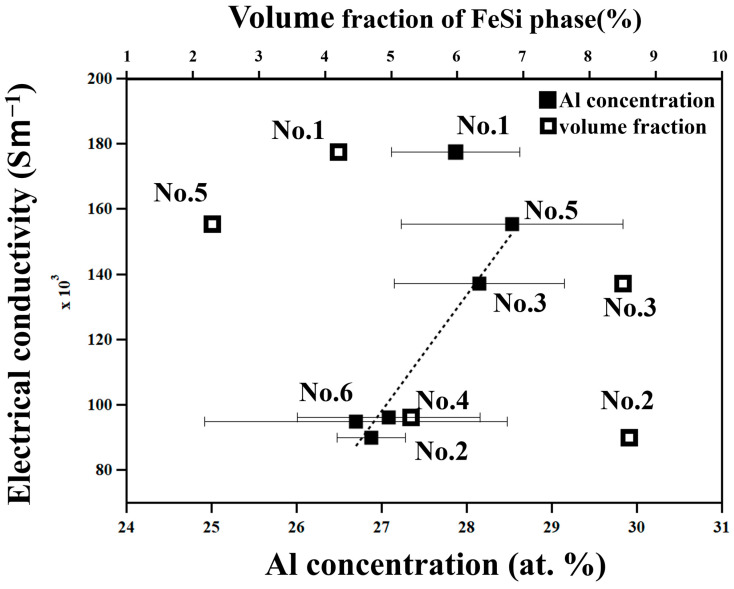
Electrical conductivity, σ, at 322 K of all the samples in this work plotted as a function of the Al concentration of the t_1_-Al_2_Fe_3_S_3_ phase determined by EDS and volume fraction of the ε-FeSi phase determined by image analysis.

Samples with higher σ have lower *S*. This suggests that carrier concentration is the dominant factor affecting the difference in σ and *S* among samples. The τ_1_-Al_2_Fe_3_Si_3_ phase has a composition range with variable Al/Si ratios from 21 at. % Al to 41.5 at. % Al [20]. As reported in previous works [19,26], the Al/Si ratio determines the carrier concentration since excess Al and Si atoms generate holes and electrons, respectively. Actually, σ increases with increasing Al concentrations in the τ_1_ phase, as shown in Figure 7.

The error bars for the Al concentration in Figure 7 might be too large, as evaluated by numerical values and shown in Table 2, to discuss such a trend between the electrical conductivity and the Al concentration. Therefore, to check the validity of the aluminum concentration for all the samples, we determined the lattice parameters of each sample by the Rietveld analysis using the Rigaku-PDXL software (version 2.7.2.0) and show the results in Figure S2. The lattice parameters *a*, α, β, and γ are in good agreement with those reported in a previous work [20]. The result of the lattice parameters of *b* and *c* does not show a clear dependence on Al concentration. This is possibly due to the overlap of peaks between the ε-FeSi phase and τ_1_-Al_2_Fe_3_Si_3_ phase. Thus, from another point of view, the ε-FeSi phase has high σ [27] and might affect σ via the rule of mixtures. Therefore, we evaluated the volume fraction of the ε-FeSi phase by image analysis using the ImageJ software, and s is plotted as a function of it in Figure 7. As a result, it is found that s depends on the Al concentration rather than the volume fraction of the ε-FeSi phase because the volume fraction of the ε-FeSi phase is less than 10% in all samples, which is too small to show a significant effect on σ. Focusing on the carrier transport across the interfaces between the ε-FeSi phase and τ_1_-Al_2_Fe_3_Si_3_ phase, it has been reported [23] that they are of the ohmic-type, that is, they do not show an energy barrier and hence do not work as resistance for passing carriers.

The exceptionally high conductivity of the No. 1 sample, which was prepared by reactive sintering (Table 1), can be attributed to the high mobility of carriers since nanometer-scale features are not observed in this sample only.

**Figure 8:**
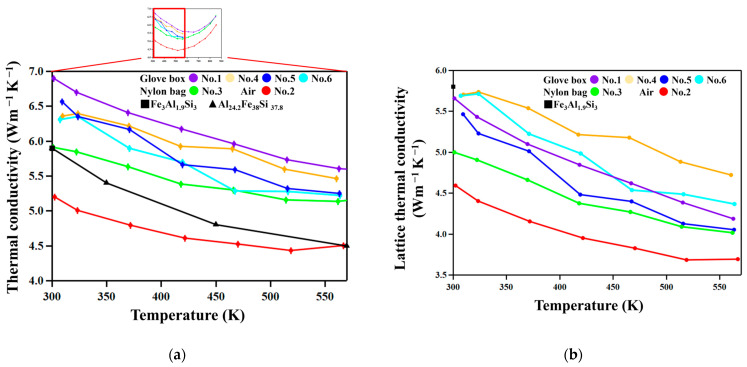
(**a**) The thermal conductivities as functions of temperature. The thermal conductivity of Fe_3_Al_1.9_Si_3_ from reference [23] and Al_24.2_Fe_38_Si_37.8_ from reference [26]. (**b**) The lattice thermal conductivities as functions of temperature. The lattice thermal conductivity of Fe_3_Al_1.9_Si_3_ from reference [23].

Figure 8 shows the temperature dependence of the total and lattice thermal conductivities, κ_tot_ and κ_lat_, respectively. κ_lat_ was calculated using the Wiedemann–Franz law, κ_tot_–*L*σ*T*, where *L* is the Lorenz number [28]. We used an approximate expression for *L* for degenerate semiconductors [29], *L* = 1.5 + exp[−|*S*|/116] × 10^−8^ WΩK^−2^, where *S* is in µVK^−1^. No clear trend was recognized in the relation between chemical composition and κ_tot_ and κ_lat_. On the other hand, samples prepared in nylon bags (No. 2 and 3) are lower than those prepared using the glove box.

**Figure 9:**
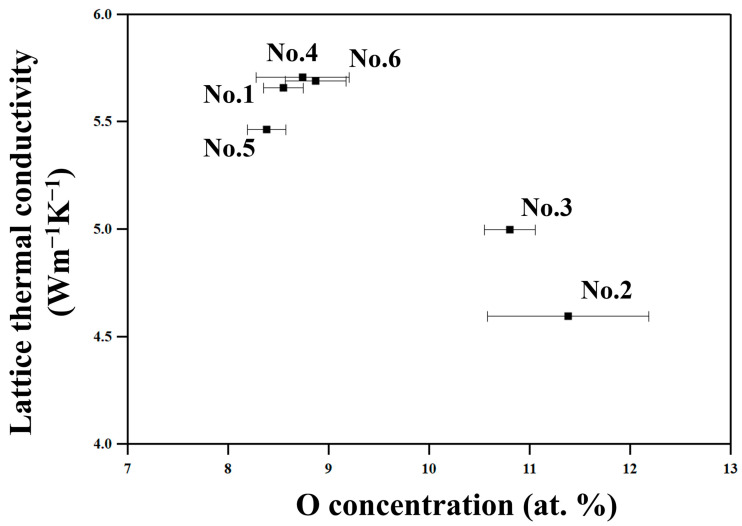
The lattice thermal conductivities at 322 K of all the samples in this work plotted as a function of the O concentration determined by EDS.

The No. 2 and 3 samples, which are milled in environments where samples are relatively easily oxidized and hence have higher oxygen concentrations (Table 2), show lower κ_tot_ and κ_lat_ than the other samples, as Figure 9 shows. This suggests that the oxide particles in nanometer scales effectively scatter phonons, resulting in such decreased thermal conductivities. On the other hand, the No. 1, 4, 5, and 6 samples show relatively high κ_tot_ and κ_lat_ since they were milled in an atmosphere with lower oxygen partial pressure in a glove box equipped with a gas circulation purification system, and thus have less oxide particles than samples No. 2 and 3.

We compare here the thermal conductivities of the FAST materials studied in this work with those of the FAST materials in the literature. As seen in Figure 8b, the total thermal conductivity of sample No. 2 is lower than those reported previously for Fe_3_Al_1.9_Si_3_ [23] and Al_24.3_Fe_38_Si_37.8_ [26] compositions, but others are not. The reason is that the total thermal conductivity is contributed to by electrons and it should be significantly dependent on composition. Therefore, to unveil the effect of nanostructuring, it would be more suitable to look into the lattice aspect of thermal conductivity shown in Figure 8b. As found in the figure, the lattice thermal conductivities of the No. 2 and 3 samples are significantly lower than that of Fe_3_Al_1.9_Si_3_ [23], which is not nanostructured, due to the scattering of phonons by oxide particles.

Thus, nanostructuring effectively reduces lattice thermal conductivity by particles of various sizes. A theoretical calculation for FAST materials based on first principles [30] have revealed that the mean free path of phonons contributing to κ is below 500 nm and significant contribution is between 3 nm to 200 nm. Thus, particles size is important for reduced lattice thermal conductivity to be recognized. The SEM (Figure 5) and EBSD (Figure S4) images show that while the sizes of grains of the τ_1_Al_2_Fe_3_Si_3_ phase are of ~800 nm and 74% of the ε-FeSi particles are above 200 nm, intergranular and intragranular oxide phases are of tens of nanometers and of singlenanometer-size scales, respectively. According to the Debye–Callaway model [31], the relaxation time for phonon scattering is expressed as the summation of relaxation time by various scattering modes, *τ*^−1^ = *τ*_D_^−1^ + *τ*_P_^−1^ + *τ*_B_^−1^, where *τ*_D_^−1^, *τ*_P_^−1^, and *τ*_B_^−1^ are relaxation times for point-defect scattering, phonon–phonon scattering, and boundary scattering, respectively. Among these scattering modes, boundary scattering is dominant at low temperatures [31].

The relaxation time of boundary scattering is expressed as *τ*_B_^−1^ *= υ*_s_*/L*, where *υ*_s_ is the velocity of sound and *L* is a characteristic length. Oxide particles effectively reduce the *L* of the matrix, resulting in a reduced relaxation time of boundary scattering, and effectively scatter phonons, resulting in a decreased thermal conductivity.

We checked the thermal stability of the nanostructures by annealing samples for one week in a vacuum at 573 K followed by microstructure observations and thermal diffusivity measurement. Regarding the results, the thermal diffusivity and oxide particle size are not changed by the annealing, as Figure S6 shows.

**Figure 10:**
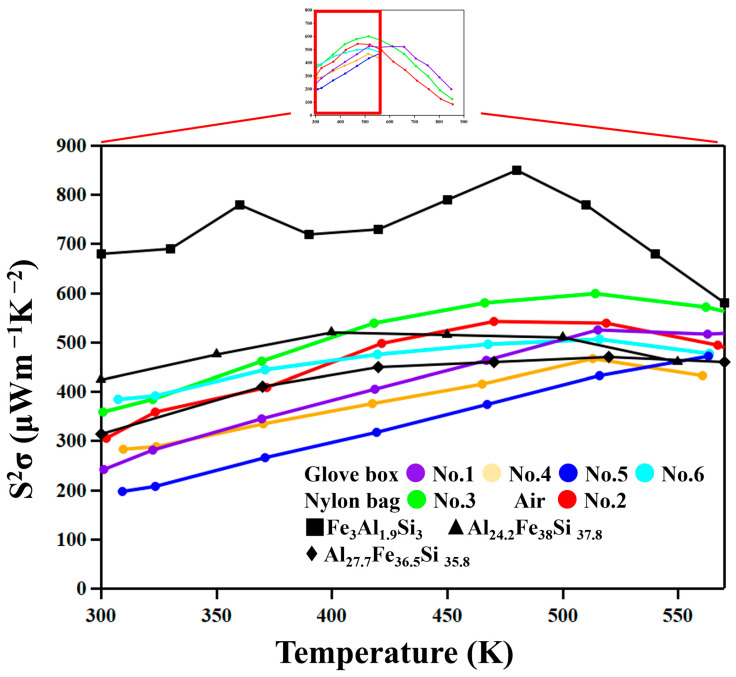
Power factors as functions of temperature. The Power factors of Fe_3_Al_1.9_Si_3_ from reference [23], Al_24.2_Fe_38_Si_37.8_ from reference [26] and Al_27.7_Fe_36.5_Si_35.8_ from reference [19].

Figure 10 shows the temperature dependence of the power factor, *PF* = *S*^2^σ. The *PF* of the No. 2 and 3 samples, which show low thermal conductivity due to the existence of nanoscale oxide particles, do not show significantly lower values compared with other samples. It is thus suggested that nanostructuring does not significantly reduce the mobility of carriers in these materials.

The temperature dependence of *zT* is shown in Figure S7. As seen in this figure, *zT* values of the samples prepared by milling in a nylon bag (No. 3) and in air (No. 2), which have higher densities of oxide particles, are higher than others. This results from the relatively lower lattice thermal conductivities compared with other samples, as shown in Figure 8. Thus, the effect of nanostructuring is positive.

## 4. Conclusions

High-energy ball milling is an effective method for fabricating nanostructured Al_2_Fe_3_Si_3_ compounds and leads to a reduced lattice thermal conductivity. Nanostructuring includes the formation of oxide particles, which can be controlled via controlling oxygen partial pressure in milling, with nanometer-size scales in addition to a fine-grain structure and two-phase structure between Al_2_Fe_3_Si_3_ and its equilibrium phase. The oxide particles have been found to have the finest grain structure and to show significant effects on the reduction in lattice thermal conductivity due to enhanced phonon scattering. The thermal conductivity of ~5 WK^−1^m^−1^ at room temperature has been achieved, corresponding to a 25% reduction in thermal conductivity in the p-type Al_2_Fe_3_Si_3_ compound at room temperature.

## Figures and Tables

**Table 1:** Synthesis conditions of the samples used in this work.

Sample	Charging Composition	Excess Al (%)	Bulk Density (g/cm^3^)	Preparation Method	Milling Machine	Atmosphere in Milling/Mixing ^†^
No. 1	Al_25.7_Fe_37.1_Si_37.1_	4.0	4.99	Reactive sintering	Planetary mill	Glove box (Ar)
No. 2	Al_25.7_Fe_37.1_Si_37.1_	4.0	4.97	Mechanical alloying	Spex mill	Air
No. 3	Al_25.7_Fe_37.1_Si_37.1_	4.0	5.03			Sealed with Ar gas in a nylon bag
No. 4	Al_25.7_Fe_37.1_Si_37.1_	4.0	5.00			Glove box (Ar)
No. 5	Al_26.8_Fe_36.6_Si_36.6_	10.0	5.12			
No. 6	Al_27.5_Fe_36.2_Si_36.2_	14.0	4.96			

^†^ In all cases, samples were packed with Ar gas in a ZrO_2_ or WC vial with a clamping screw and an O-ring, as the packing was not necessarily perfect under the hard-vibrating conditions during milling for long periods of time. The “Atmosphere” in the table means the atmosphere surrounding the vial.

**Table 2:** Chemical compositions of the respective phases measured by EDS.

Sample	Phase	Al (at. %)	Fe (at. %)	Si (at. %)	O (at. %) ^#1^
No. 1	τ_1_-Al_2_Fe_3_Si_3_	27.87 ±0.75	39.30 ±0.47	32.50 ±0.57	8.55 ±0.20
	ε-FeSi	11.37 ±0.67	47.82 ±0.32	40.34 ±0.48	
No. 2	τ_1_-Al_2_Fe_3_Si_3_	26.88 ±1.30	38.01 ±0.48	35.12 ±1.15	11.38 ±0.80
	ε-FeSi ^#2^	-	-	-	
No. 3	τ_1_-Al_2_Fe_3_Si_3_	28.15 ±1.00	37.00 ±0.40	34.85 ±0.75	10.80 ±0.25
	ε-FeSi ^#2^	-	-	-	
No. 4	τ_1_-Al_2_Fe_3_Si_3_	27.08 ±1.07	37.25 ±1.15	35.67 ±1.40	8.74 ±0.46
	ε-FeSi ^#2^	-	-	-	
No. 5	τ_1_-Al_2_Fe_3_Si_3_	28.53 ±1.78	37.02 ±1.39	34.45 ±1.94	8.38 ±0.19
	ε-FeSi ^#2^	-	-	-	
No. 6	τ_1_-Al_2_Fe_3_Si_3_	26.7 ±0.4	38.23 ±0.27	35.03 ±0.44	8.87 ±0.30
	τ_8_-Al_2_Fe_3_Si_4_^#2^	-	-	-	

^#1^ Oxygen concentration was measured as an average from large areas containing all constituent phases in each sample. The Al, Fe, and Si concentrations were measured as point scans without oxygen concentration. See the main text for details. ^#2^ The second phase is too small to be analyzed for its accurate chemical composition.

## Data Availability

The original contributions presented in this study are included in the article/Supplementary Materials. Further inquiries can be directed to the corresponding author.

## Supplementary Materials

The following supporting information can be downloaded at https://www.mdpi.com/article/10.3390/ma18225193/s1. Figure S1: Lattice backscattered electron (BSE) images of sample No. 1. Figure S2: Lattice parameters obtained from the samples used in this study plotted as a function of Al concentration together with the literature data [20]. Figure S3: The result of the Rietveld analysis of the No. 3 sample using the Rigaku-PDXL software. Since there are many peaks from the τ_1_-Al_2_Fe_3_Si_3_ phase because of its low symmetric crystal structure, some of the ε-FeSi phase peaks overlap with those of the τ_1_-Al_2_Fe_3_Si_3_ phase. Examples include the peak of the ε-FeSi (2 1 1) plane overlapping with that of the τ_1_-Al_2_Fe_3_Si_3_ (2 −2 −2) plane at nearly 49°, as seen in the small window. Figure S4: Phase map obtained from electron backscatter diffraction pattern (EBSD) analysis of samples No. 1 (a) and No. 3 (b) after sintering. The red region is the τ_1_-Al_2_Fe_3_Si_3_ phase. The green region is the e-FeSi phase. Figure S5: The grain size distribution of the FeSi phase in the No. 3 sample. The mean grain size ($\bar{d}$) is 226 nm. The volume fraction here shows the ratio of the volume of FeSi particles within each size range to the total volume of the FeSi phase. Figure S6: The thermal diffusivity of the No. 3 sample as a function of temperature. Figure S7: *zT* values of all samples as functions of temperature.

## References

[1] Sudevalayam S., Kulkarni P. (2011). Energy Harvesting Sensor Nodes: Survey and Implications. IEEE Commun. Surv. Tutor..

[2] Takagiwa Y., Hou Z., Tsuda K., Ikeda T., Kojima H. (2021). Fe-Al-Si Thermoelectric (FAST) Materials and Modules: Diffusion Couple and Machine-Learning-Assisted Materials Development. ACS Appl. Mater. Interfaces.

[3] Takagiwa Y., Shinohara Y. (2019). A practical appraisal of thermoelectric materials for use in an autonomous power supply. Scr. Mater..

[4] Snyder G.J., Snyder A.H. (2017). Figure of merit ZT of a thermoelectric device defined from materials properties. Energy Environ. Sci..

[5] Venkatasubramanian R., Siivola E., Colpitts T., O’Quinn B. (2001). Thin-film thermoelectric devices with high room-temperature figures of merit. Nature.

[6] Wei J., Yang L., Ma Z., Song P., Zhang M., Ma J., Yang F., Wang X. (2020). Review of current high-ZT thermoelectric materials. J. Mater. Sci..

[7] Qian X., Zhou J., Chen G. (2021). Phonon-engineered extreme thermal conductivity materials. Nat. Mater..

[8] Nomura M., Shiomi J., Shiga T., Anufriev R. (2018). Thermal phonon engineering by tailored nanostructures. Jpn. J. Appl. Phys..

[9] Kanatzidis M.G. (2010). Nanostructured Thermoelectrics: The New Paradigm?. Chem. Mater..

[10] Masoumi S., Pakdel A. (2021). Nanoengineering Approaches to Tune Thermal and Electrical Conductivity of a BiSbTe Thermoelectric Alloy. Adv. Eng. Mater..

[11] Maranets T., Cui H., Wang Y. (2023). Lattice thermal conductivity of embedded nanoparticle composites: The role of particle size distribution. Nanotechnology.

[12] He Q., Yang D., Xia S., Song H. (2024). Ultra-low thermal conductivity and improved thermoelectric performance in La2O3-dispersed Bi2Sr2Co2Oy ceramics. Mater. Sci. Eng. B.

[13] Chen G., Dames C., Rowe D. (2005). Thermal Conductivity of Nanostructured Thermoelectric Materials. Thermoelectrics Handbook: Macro to Nano.

[14] Biswas K., He J., Blum I.D., Wu C.-I., Hogan T.P., Seidman D.N., Dravid V.P., Kanatzidis M.G. (2012). High-performance bulk thermoelectrics with all-scale hierarchical architectures. Nature.

[15] Pardavi-Horvath M., Takacs L. (1992). Iron-alumina nanocomposites prepared by ball milling. IEEE Trans. Magn..

[16] Alinejad B., Ikeda T. (2019). Low temperature rapid fabrication of magnesium silicide for thermoelectric application. Funct. Mater. Lett..

[17] Novák P., Michalcová A., Voděrová M., Šíma M., Šerák J., Vojtěch D., Wienerová K. (2010). Effect of reactive sintering conditions on microstructure of Fe–Al–Si alloys. J. Alloys Compd..

[18] Shiota Y., Muta H., Yamamoto K., Ohishi Y., Kurosaki K., Yamanaka S. (2017). A new semiconductor Al 2 Fe 3 Si 3 with complex crystal structure. Intermetallics.

[19] Takagiwa Y., Isoda Y., Goto M., Shinohara Y. (2018). Conduction type control and power factor enhancement of the thermoelectric material Al2Fe3Si3. J. Phys. Chem. Solids.

[20] Marker M.C., Skolyszewska-Kuhberger B., Effenberger H.S., Schmetterer C., Richter K.W. (2011). Phase equilibria and structural investigations in the system Al-Fe-Si. Intermetallics.

[21] DiTusa J.F., Friemelt K., Bucher E., Aeppli G., Ramirez A.P. (1998). Heavy fermion metal—Kondo insulator transition in FeSi1-xAlx. Phys. Rev. B.

[22] Ikeda T. High-Throughput Phase Diagram Examinations on Multicomponent Systems Using Sintered Diffusion Multiples. In Proceeding of the 11th Pacific Rim International Conference on Advanced Materials and Processing, ICC.

[23] Srinithi A.K., Sepehri-Amin H., Takagiwa Y., Hono K. (2022). Effect of microstructure on the electrical conductivity of p-type Fe–Al–Si thermoelectric materials. J. Alloys Compd..

[24] Ikeda T., Haviez L., Li Y., Snyder G.J. (2012). Nanostructuring of thermoelectric Mg(2) Si via a nonequilibrium intermediate state. Small.

[25] Goldstein J., Newbury D.E., Joy D.C., Lyman C.E., Echlin P., Lifshin E., Sawyer L., Michael J.R. (2003). Scanning Electron Microscopy and X-Ray Microanalysis.

[26] Takagiwa Y., Ikeda T., Kojima H. (2020). Earth-Abundant Fe-Al-Si Thermoelectric (FAST) Materials: From Fundamental Materials Research to Module Development. ACS Appl. Mater. Interfaces.

[27] Sales B.C., Jones E.C., Chakoumakos B.C., Fernandez-Baca J.A., Harmon H.E., Sharp J.W., Volckmann E.H. (1994). Magnetic, transport, and structural properties of Fe1-xIrxSi. Phys. Rev. B Condens. Matter..

[28] Asai J., Bumrungpon M., Tsubochi T., Kanaya T., Tachii M., Maeda T., Hasezaki K. (2021). Shift of tellurium solid-solubility limit and enhanced thermoelectric performance of bismuth antimony telluride milled with yttria-stabilized zirconia balls and vessels. J. Eur. Ceram. Soc..

[29] Kim H.-S., Gibbs Z.M., Tang Y., Wang H., Snyder G.J. (2015). Characterization of Lorenz number with Seebeck coefficient measurement. APL Mater..

[30] Sato N., Takagiwa Y. (2021). First-Principles Study on Lattice Dynamics and Thermal Conductivity of Thermoelectric Intermetallics Fe3Al2Si3. Crystals.

[31] Callaway J., von Baeyer H.C. (1960). Effect of Point Imperfections on Lattice Thermal Conductivity. Phys. Rev..

