# Supplementary material for: Characterization and Preparation of Nanostructured Al2Fe3Si3 Thermoelectric Materials
Source: Materials (Basel). 2025 Nov 15;18(22):5193. doi: 10.3390/ma18225193 (PMC12654535; doi:10.3390/ma18225193)
Supplement: Supplementary file 1 [file materials-18-05193-s001.zip › materials-3952993-supplementary.pdf]

Supplementary materials

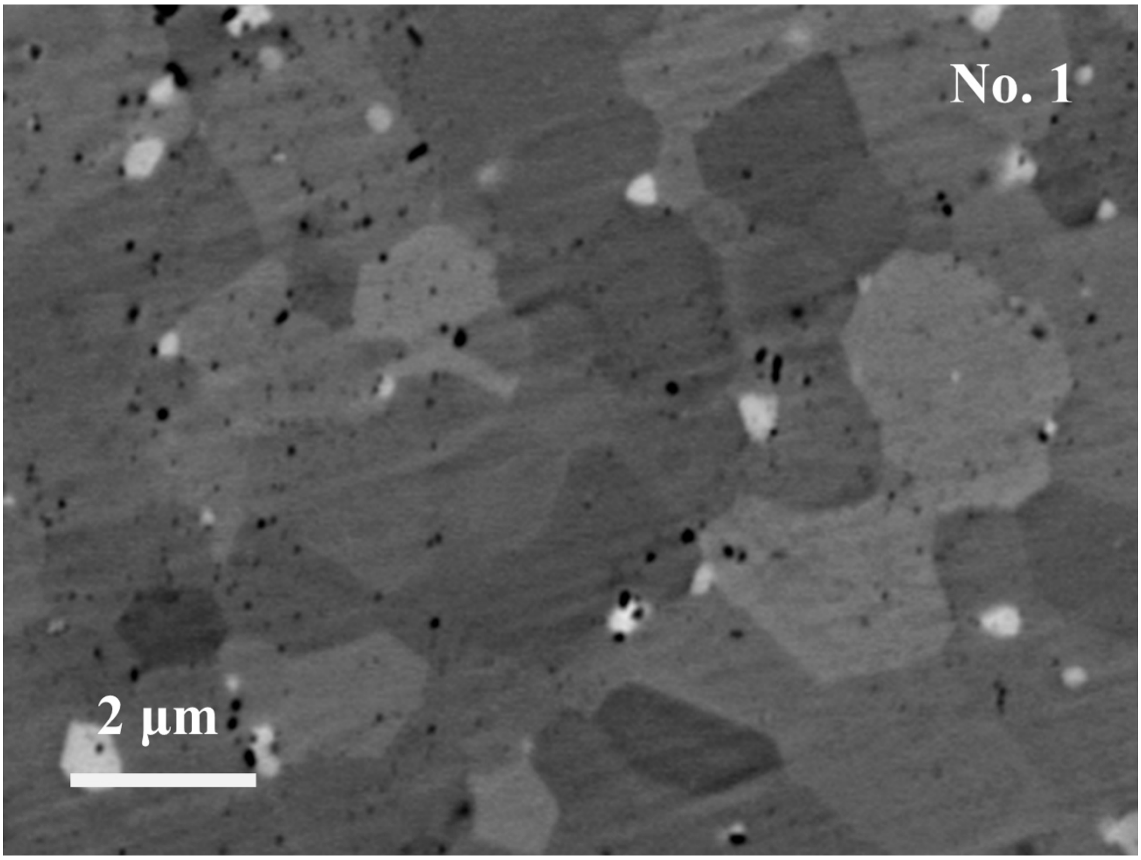

Figure S1. Lattice Backscattered electron (BSE) images of samples No. 1

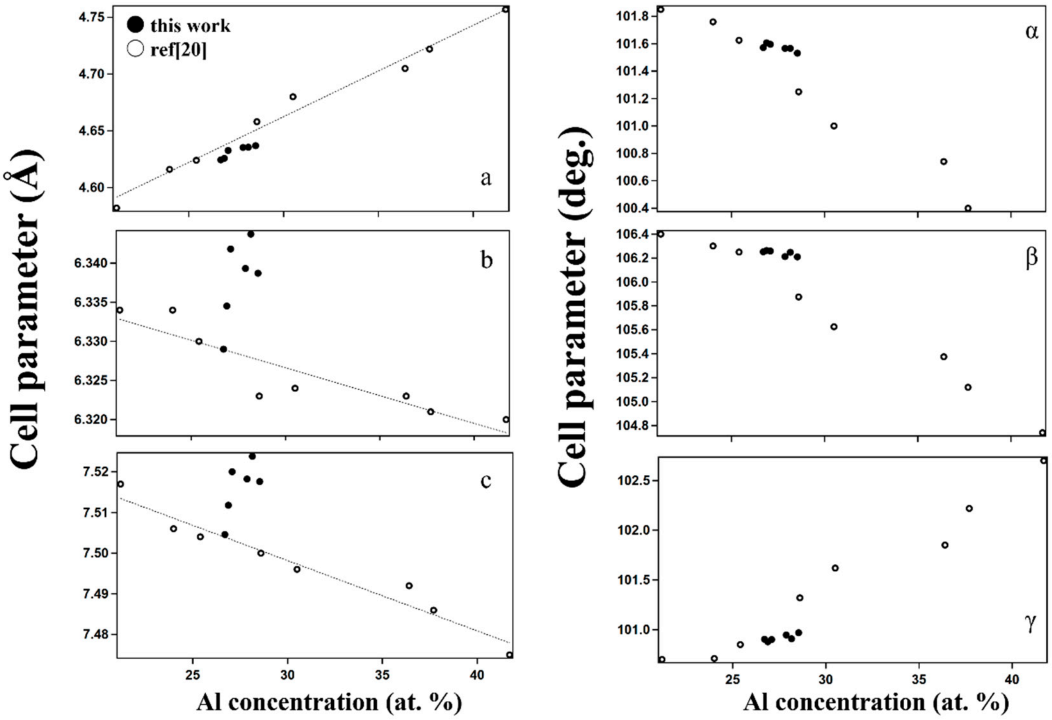

Figure S2. Lattice parameters obtained from the samples used in this study plotted as a functeion of Al concentration together with the literature data [20].

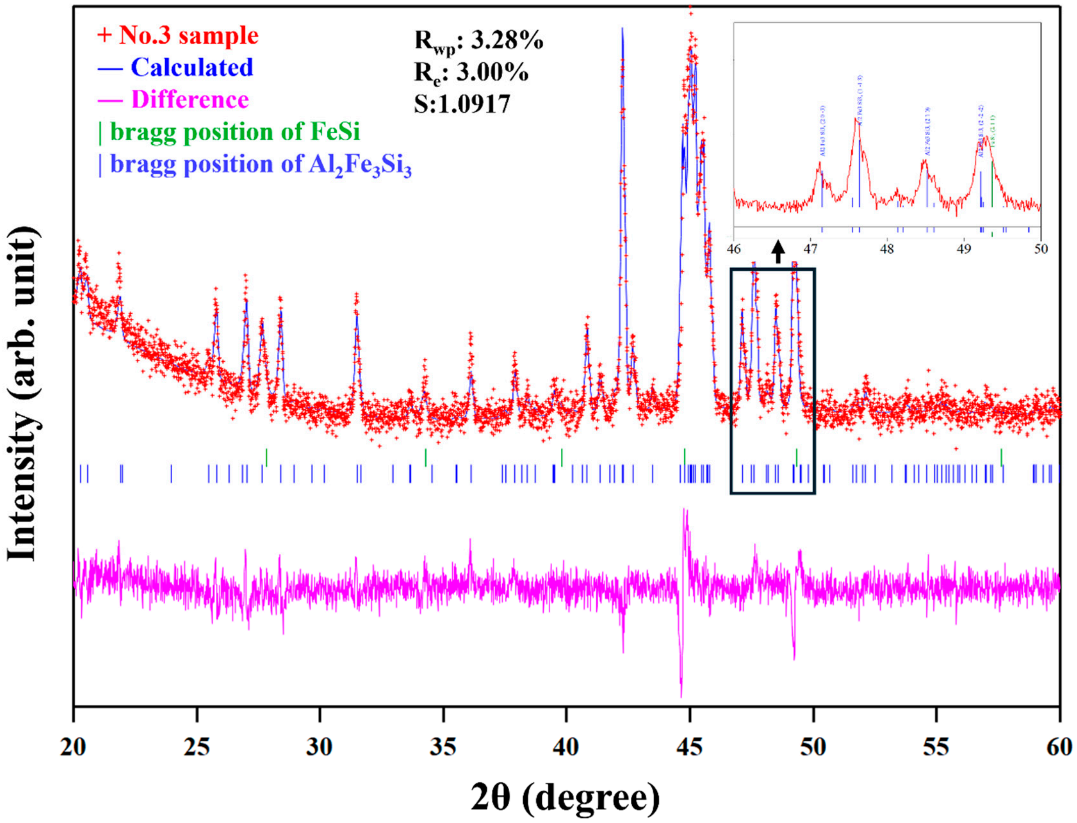

Figure S3. Result of the Rietveld analysis of the No.3 samples using the Rigaku-PDXL software. Since there are many peaks from the τ_1-$Al_2Fe_3Si_3$ phase becuse of the low symmetric crystal structure of it, some of the e-FeSi phase peaks overlap with those of the τ_1-$Al_2Fe_3Si_3$ phase. Examples include the peak of the ε-FeSi (2 1 1) plane overlap with that of the τ_1-$Al_2Fe_3Si_3$ (2 −2 −2) plane nearly 49° as seen in the small window.

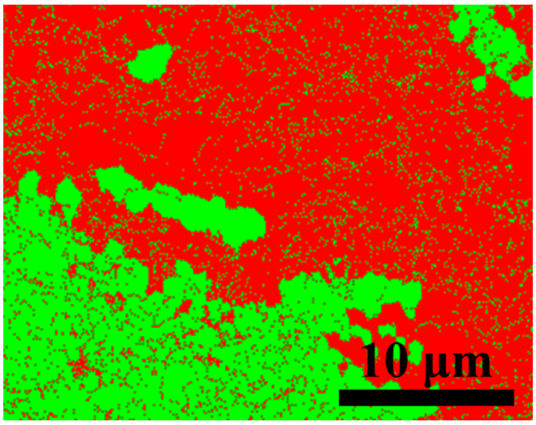

(a)

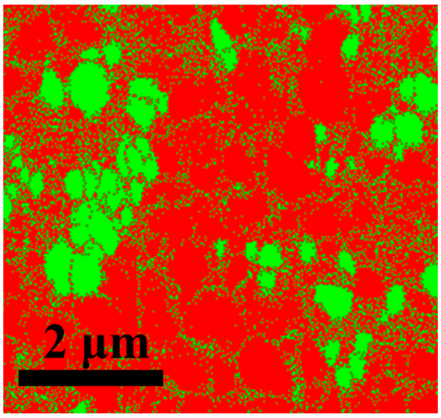

(b)

Figure S4. Phase map obtained from electron backscatter diffraction pattern (EBSD) analysis of samples No. 1 (a) and No. 3 (b) after sintering. The red region is τ_1-$Al_2Fe_3Si_3$ phase. The green region is ε-FeSi phase

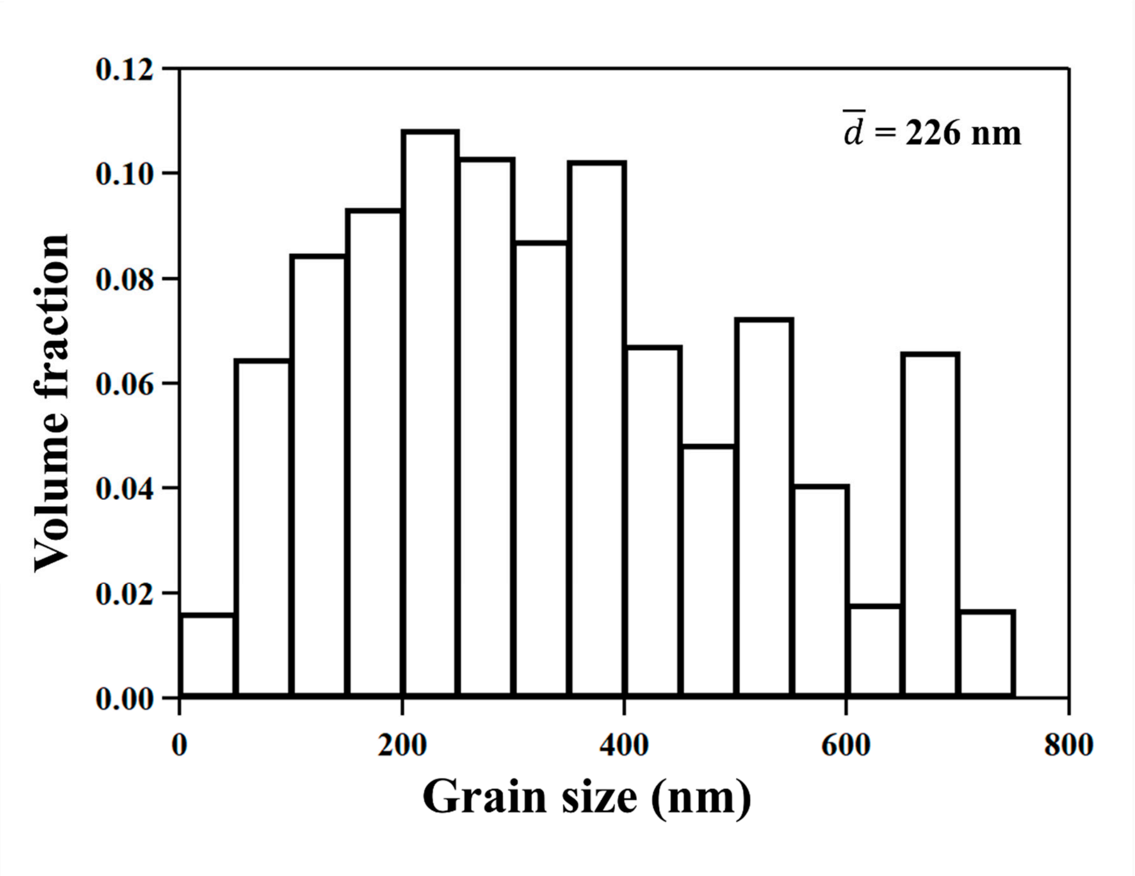

Figure S5. The grain size distribution of FeSi phase in No. 3 sample. The mean grain size ($\bar{d}$) is 226 nm. The volume fraction here shows the ratio of the volume of FeSi particles within each size range to the total volume of the FeSi phase.

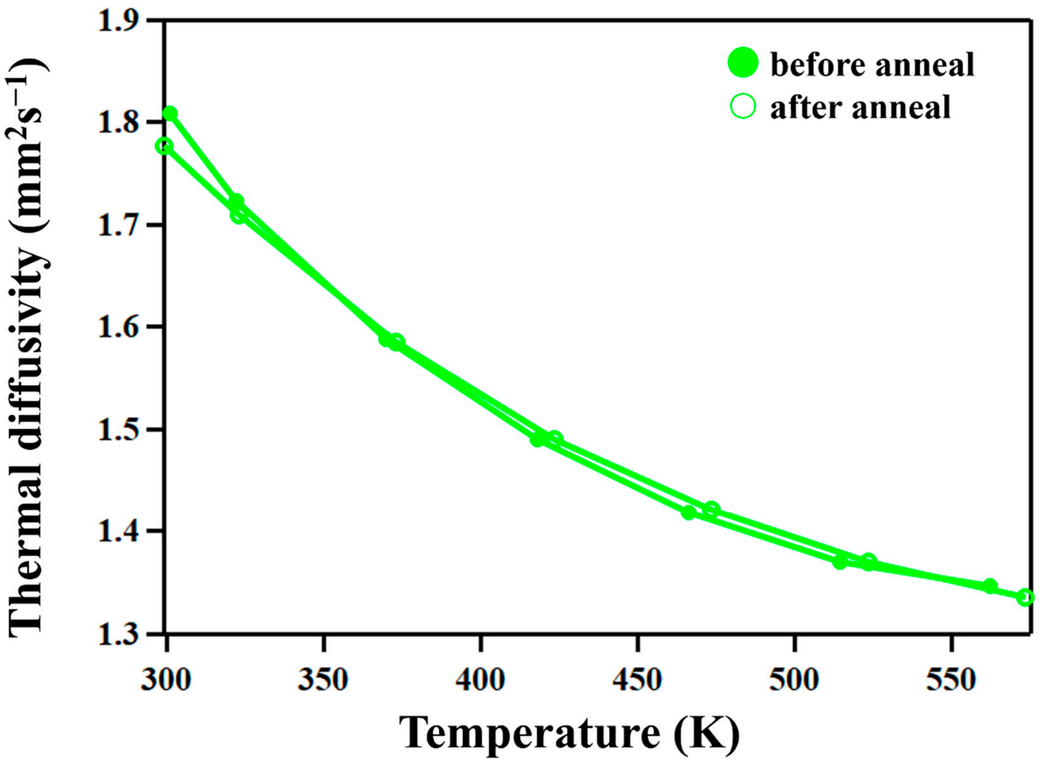

Figure S6. Thermal diffusivity of No.3 sample as functions of temperature.

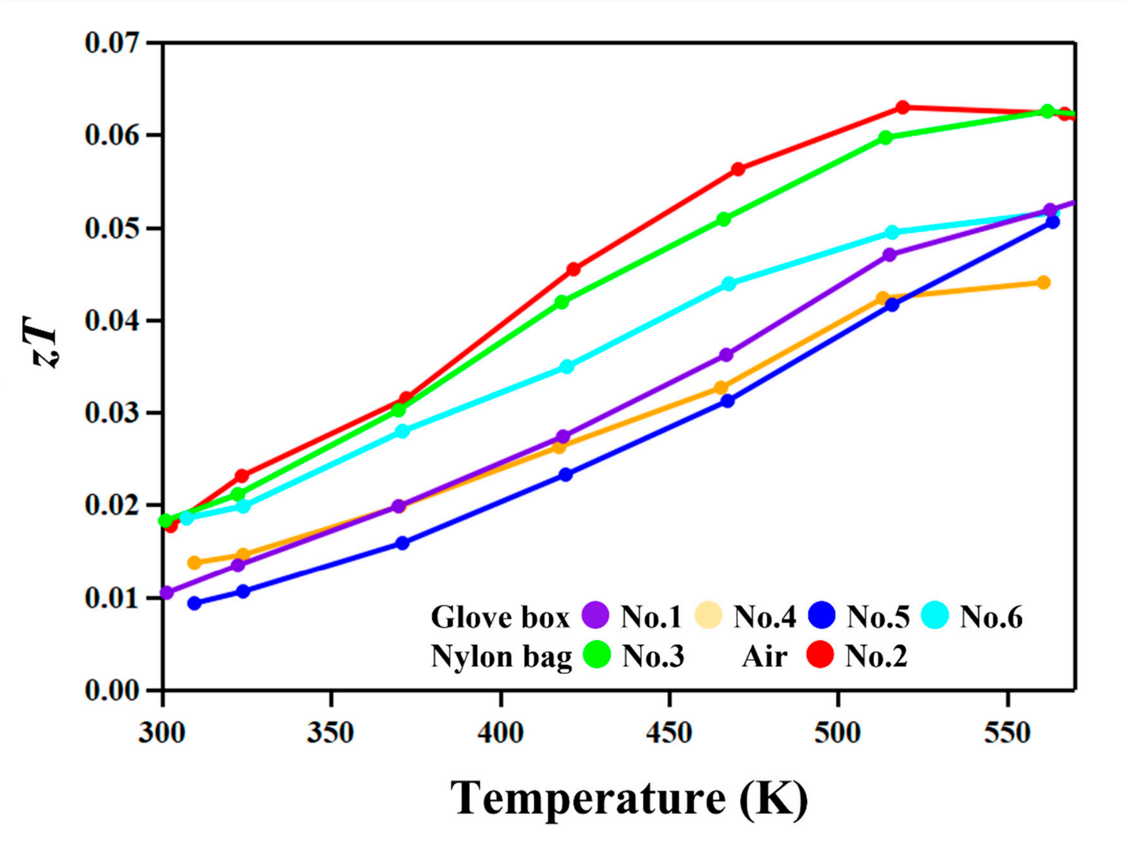

Figure S7. *zT* values of all samples as functions of temperature.
